# The effectiveness of psychosocial interventions for anxiety in children and adolescents with autism spectrum disorder: a systematic review and meta-analysis

**DOI:** 10.1186/s13034-015-0054-7

**Published:** 2015-06-20

**Authors:** Ance Kreslins, Ashley E. Robertson, Craig Melville

**Affiliations:** Institute of Health and Wellbeing, University of Glasgow, 1st Floor Admin Building Gartnavel Royal Hospital, 1055 Great Western Road, Glasgow, G12 0XH, Scotland

**Keywords:** Meta-analysis, Autism spectrum disorder/ASD, Anxiety, Child, Adolescent, Psychosocial intervention, Cognitive behavioural therapy/CBT

## Abstract

Anxiety is a common problem in children and adolescents with autism spectrum disorder (ASD). This meta-analysis aimed to systematically evaluate the evidence for the use of psychosocial interventions to manage anxiety in this population. Cognitive behavioural therapy (CBT) was the primary intervention modality studied. A comprehensive systematic search and study selection process was conducted. Separate statistical analyses were carried out for clinician-, parent-, and self-reported outcome measures. Sensitivity analyses were conducted by removing any outlying studies and any studies that did not use a CBT intervention. A subgroup analysis was performed to compare individual and group delivery of treatment. Ten randomised control trials involving a total of 470 participants were included. The overall SMD was d = 1.05 (95 % CI 0.45, 1.65; z = 3.45, p = 0.0006) for clinician- reported outcome measures; d = 1.00 (95%CI 0.21, 1.80; z = 2.47, p = 0.01) for parent-reported outcome measures; and d = 0.65 (95%CI -0.10, 1.07; z = 1.63, p = 0.10) for self-reported outcome measures. Clinician- and parent-reported outcome measures showed that psychosocial interventions were superior to waitlist and treatment-as-usual control conditions at post-treatment. However, the results of self-reported outcome measures failed to reach significance. The sensitivity analyses did not significantly change these results and the subgroup analysis indicated that individual treatment was more effective than group treatment. The main limitations of this review were the small number of included studies as well as the clinical and methodological variability between studies.

## Introduction

Autism Spectrum Disorder (ASD) is a neurodevelopmental disorder characterised by a triad of symptoms – deficits in social communication, impaired social interaction and lack of flexibility of thought and behaviour. These features appear in early childhood and endure across the lifespan [1]. ASD is an umbrella term introduced in the DSM-5 to define a continuum of symptoms, formerly classified as separate autistic disorders. These disorders included Autistic disorder, Asperger syndrome (AS), and pervasive developmental disorder not otherwise specified (PDD-NOS) [2]. ASD has a prevalence of 1.16 % in the general population [3].

Around 70 % of children with ASD also experience psychiatric comorbidity [4], with one of the most common being anxiety [5]. Although anxiety in ASD is present across the whole spectrum of the disorder, the presentation seems to be affected by individual factors, such as age, degree of social impairment and level of cognitive functioning [6]. Prevalence rates of anxiety disorders in typically developing children range between 2 and 27 % [7], whereas rates of 11–84 % have been reported in the children with ASD [6]. However, it should be noted that rates vary substantially between studies due to varying sampling methods and anxiety assessments [6]. The most commonly reported anxiety disorders in the paediatric ASD population are specific phobia (30 %), obsessive compulsive disorder (OCD) (17 %) and social anxiety (17 %) [8]. This distribution is similar to that seen in typically developing children, apart from OCD, which is more common in ASD [8, 9].

Due to a unique interaction between anxiety and core ASD symptomology, the manifestation of anxiety in children and adolescents with ASD differs in several ways from anxiety seen in typically developing youth [10]. Anxiety in ASD is associated with more behavioural problems, such as social avoidance, repetitive behaviours and aggression [6, 11]. These maladaptive behaviours may be difficult to differentiate from symptoms of ASD [6, 9, 12] resulting in anxiety being underreported in this population [13, 14]. As a result of the significant diagnostic overlap between anxiety disorders and core ASD symptoms, it has been discussed whether anxiety should be considered as part of ASD [8, 11]. However, ASD may simply predispose to anxiety [9] since individuals with ASD struggle to manage perceived threatening external stimuli due to deficits in executive functioning, inherent difficulties understanding emotions, and problems with social and communication skills [9, 11, 15]. Furthermore, anxiety levels in ASD youth may be affected by an increased or decreased sensitivity to sensory stimuli and motor clumsiness [15].

Cognitive behavioural therapy (CBT) has been shown to be effective in treating anxiety in typically developing children and adolescents [16]. Furthermore, CBT can be modified in a number of ways to make this treatment modality more suitable for the ASD population [15]. Some of the issues that may affect the delivery of CBT in ASD youth are difficulties responding to social cues and engaging in reciprocal exchanges [17], as well as reduced verbal skills and difficulties processing figurative meaning [15]. In addition, ASD youth may have difficulties understanding and expressing emotions, and may have reduced Theory of Mind (ToM) abilities, i.e. the ability to identify their own and other individuals’ thoughts and emotions [18, 19]. It is essential that therapists have insight into the difficulties people with ASD may face in order to develop a therapeutic content, setting and relationship that is tailored specifically for the needs of this population [15].

The majority of research carried out to ascertain the effectiveness of psychosocial interventions in the ASD population is biased toward the high functioning end of the spectrum [6]. This may be due to the logistical and ethical issues that may arise when working with more severely impaired individuals, including difficulties with communication, giving informed consent, attending to tasks and following instructions. The exclusion of youths on the spectrum with cognitive limitations creates problems with generalising results to the Autism Spectrum as a whole. This needs to be taken into account when interpreting study results. In the UK, individual or group CBT is therefore recommended to manage anxiety in children and young people with ASD if they have the necessary verbal and cognitive abilities [4].

The literature addressing treatment options for anxiety in ASD youth has been constantly growing over the past decade. At present, a considerable number of studies investigating psychosocial interventions for anxiety in children and adolescents with ASD have been conducted. Furthermore, comparable outcome measures have been used in these studies, making it possible to perform a meta-analysis. The primary treatment modality studied in this meta-analysis was CBT, but it was considered important to also include other types of psychosocial modalities, such as social skills interventions, since components of these may be used to optimise content and delivery of any anxiety management intervention aimed at ASD youth.

The objective of this meta-analysis was to systematically review the evidence for the use of psychosocial interventions to manage anxiety in children and adolescents with ASD.

## Methods

### Information sources and search strategy

This review was designed in accordance with the PRISMA guidelines [20]. Two independent researchers identified studies by searching electronic databases and manually finding suitable published studies. The following databases were searched: Web of Science, PsychINFO, Embase, Medline and Cochrane Database of Systematic Reviews (Cochrane Library). The search strategy included terms such as ASD, auti*, child*, anxi*, psychotherap* and cognitive behavi* therap*. It was limited to the title and abstract or the topic, depending on the availability of search options within each database. In addition, the search was limited to journals in English with a publication year 2000–2013 due to practical reasons and the fact that, to our knowledge, there were no studies published prior to 2000 that met our inclusion criteria. The final search was run on the 13^th^ of November 2013.

### Eligibility criteria and study selection

Studies were included if they met the following criteria: a) the study was published in English and between the years 2000–2013; b) the study was a randomised control trial (RCT); c) the patient population was children and/or adolescents (age 0–18 years) with a primary diagnosis of ASD and clinically significant anxiety symptoms; and d) at least one outcome measure was a standardised continuous measure of anxiety (parent-, clinician-, or self-reported). Studies were screened based on the title and abstract. The final selection of studies was performed using tools provided in the Cochrane Collaboration Handbook [21].

### Selection of outcome measures

Outcome measures were selected depending on their validity and frequency of use. Judgement of the validity of anxiety measures in the ASD population was based on two recently published, methodologically rigorous reviews [10, 11]. The frequency of use was considered important to ensure maximum possible comparability between study results. The selected clinician-reported outcome measures were The Anxiety Disorders Interview Schedule – Child/Parent version (ADIS-C/P) [22], The Pediatric Anxiety Rating Scale (PARS) [23] and The Childhood Anxiety Sensitivity Index - Anxiety (CASI-Anx) [24]. Spence Children's Anxiety Scale – Parent version (SCAS-P) [25] and Multidimensional Anxiety Scale for Children – Parent version (MASC-P) [26] were used as parent-reported outcome measures. Children's Anxiety Scale – Child version (SCAS-C) [25], Multidimensional Anxiety Scale for Children – Child version (MASC-C) [26], Revised Children's Manifest Anxiety Scale (RCMAS) [27] and Social Interaction Anxiety Scale (SIAS) [28] were chosen as self-reported outcome measures.

If a study used two of the selected outcome measures, one of the measures was chosen for the analysis. Storch et al. [29] reported both ADIS-C/P and PARS scores. Although both measures are considered to be equally well validated [11], PARS was selected for the purpose of this review since the required ADIS-C/P scores were not available. Chalfant et al. [30] included both SCAS-C and MASC-C. The superiority of the SCAS-C or the MASC-C in terms of validity was not clear [10, 11]. Therefore, SCAS-C was chosen based on its frequency of use across the reviewed studies. Although RCMAS and SIAS had not been validated for use in the ASD population [10, 11], they were used when studies lacked results from more validated outcome measures. Storch et al. [29] did not report total RCMAS scores. Therefore, an average of the subscale scores was used.

### Data collection process and risk of bias within studies

Data extraction and risk of bias assessment was performed according to the Cochrane Collaboration Guidelines [21]. The first author conducted the systematic search and the second author verified inclusion/exclusion of a subset of studies. Both authors independently screened the originally selected studies and agreed on which studies should be selected for the review. Data extraction and risk of bias assessment were conducted independently by the first and second author. Any discrepancies between the authors’ ratings were arbitrated by an independent party. Risk of bias within studies was rated as high risk (bias that reduces reliability of results), low risk (bias that is unlikely to alter results) or unclear (bias that raises doubt about reliability of results/insufficient information provided to make judgment). Only methodological strengths and weaknesses that were relevant for the results of this meta-analysis were considered when assessing the risk of bias.

Selection bias was assessed based on adequate description of random sequence generation and concealment of treatment group allocation. Due to the nature of the interventions, blinding of participants and personnel was not feasible in any of the included studies. Therefore, all studies had a high risk of performance bias. Similarly, detection bias was high for parent- and self-reported outcome measures in all studies since blinding of these measures was not viable. The studies that blinded clinician-reported outcomes were scored as having a low risk of detection bias. Attrition bias was assessed by examining the reporting of withdrawals and drop-outs. Outcome data were considered complete if there were no missing pre- or post-treatment data, or if the study authors had carried out an intent-to-treat analysis. Protocols were not available for any of the eleven studies. Reporting bias was therefore evaluated purely based on evidence of selective outcome reporting provided in the study reports. No studies were excluded based on the risk of bias assessment.

### Summary measures and synthesis of results

Separate statistical analyses were carried out for clinician-, parent-, and self-reported outcome measures. Standardised mean difference (SMD) was used as the summary estimate of treatment effect. This summary statistic was chosen because the analysis was performed on a variety of continuous outcome measures. SMDs of -0.2,–0.5 and–0.8 were deemed to be indicative of small, moderate and large effects respectively [31]. According to the Cochrane Collaboration Guidelines [21], the SMD can be calculated using means and standard deviations either of final measurements or of changes from baseline. Standard deviations of changes were not reported in any of the included studies. Therefore, SMD estimates were calculated based on the post-treatment scores and standard deviations provided in each study report. No adjustments of the scores were required since the direction of the scales was the same for all outcome measures. The statistical significance level was set at p < 0.05. Forest plots were used to illustrate results from individual studies. In the case of multiple treatment arms, such as in the study conducted by Sung et al. [32], the average score of both intervention groups was compared to the control group score.

Higgin’s I^2^ [33] test was used to describe in percentage the impact of heterogeneity on the effect estimates. It was chosen over Cochrane’s Q Test since the latter has low power when there are few studies [34]. An I^2^ of less than 30 % was considered to indicate mild heterogeneity and one substantially higher than 50 % was thought to reflect substantial heterogeneity [33]. Considerable statistical heterogeneity was expected both due to clinical diversity (variability in the participants, interventions and outcomes) and methodological diversity (variability in study design and risk of bias). As a result, a random-effects model was chosen to estimate intervention effect (DerSimonian and Laird approach) [34]. All statistical analyses were conducted using Review Manager 5.1 software [35].

Follow-up data was examined to determine whether the effects of the treatment were maintained after the end of the intervention. When examining follow-up results, sufficient data was not available to conduct a statistical analysis. It was therefore assumed that the statistical analyses conducted in the individual studies were correct.

### Additional analyses

Due to the small number of studies in each review category, publication bias could not be assessed formally by using a funnel plot or statistical test [31, 36]. After discussion between the study authors, it was decided that studies would be considered outliers if the SMD was 3 times greater than the next highest SMD in that category. To analyse the effect outlying studies had on the summary estimates, a sensitivity analysis was conducted by removing any outlying studies in each category. Since the eligibility of non-CBT interventions for this meta-analysis was debatable, a sensitivity analysis was also performed by removing any studies that did not use a CBT intervention.

Due to the relatively limited research addressing psychosocial treatment options for anxiety in children and adolescents with ASD, it was deemed appropriate to include studies that used both individual and group interventions. To compare the effectiveness of these delivery methods, a subgroup analysis was conducted by comparing the confidence intervals of the summary estimates in the two subgroups (individual +/- group therapy versus group therapy only). No or minimal overlap between the confidence intervals was considered indicative of statistical significance. Subgroup analyses were only performed on outcome measures if the overall summary estimate was significant. The effects of other treatment moderators, such as age, anxiety diagnosis, parental involvement and types of intervention modifications, could not be performed due to difficulty obtaining sufficiently comparable data from the study reports.

## Results

### Study selection

**Fig. 1:**
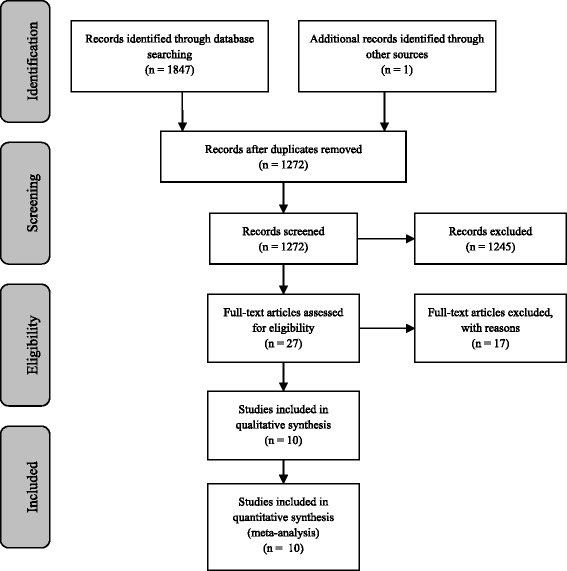
PRISMA flow diagram of study selection

The search of the databases yielded 1847 results, 575 of which were duplicates. One paper was added which had not yet been published at the time of the search [37]. 1272 records were screened based on the title and abstract, 1242 of which were excluded. An additional three were excluded due to a lack of full-text availability. Twenty-seven full-text papers were assessed for eligibility. A total of ten studies met the inclusion criteria for this review. A flow diagram of the study selection is shown in Fig. 1.

#### Study characteristics

A total of 470 (393 male, 72 female and five not reported) participants aged 7–17 years were included. Nine studies used a modified CBT intervention [29, 30, 32, 37–42] and Schohl et al. used a social skills intervention [43]. Chalfant et al., McConachie et al., Schohl et al., Sofronoff et al., and Sung et al. delivered the intervention in a group format (group sizes varying between 3 and 10 participants/group) [30, 32, 37, 40, 43]. McNally Keehn et al., Storch et al., and Wood et al. used individual therapy [29, 38, 42]. Reaven et al. and White et al. used a combination of individual and group therapy [39, 41]. The duration of the interventions varied between 6 and 16 sessions and the length of each session was 60 to 120 min.

Sung et al. [40] had an active control group in the form of a social recreational group program. The rest of the studies had waitlist or treatment-as-usual (WL/TAU) control conditions [29, 30, 32, 37, 38, 40–43]. In the majority of the included studies, participants randomised to the intervention, active control and/or WL/TAU conditions were permitted to initiate and/or continue receiving pharmacological-, psychoeducational- and/or other interventions during the study period [29, 30, 37–41]. Schohl et al., Sofronoff et al., and Wood et al. provided no information about the additional interventions received by participants. Conclusions about the extent to which these supplementary interventions may have affected the treatment effect could not be made due to insufficient and inconsistent provision of information across studies about the uptake of additional services.

The study authors established the ASD diagnoses either by using The Autism Diagnostic Observation Schedule (ADOS) [44] or by relying on a diagnosis made by a paediatrician, psychiatrist or clinical psychologist. Since the psychosocial interventions used in all ten studies required verbal communication skills, it can be assumed that a certain level of language and cognitive ability was necessary for participation in all included studies. In six studies, the presence of an anxiety disorder was required for inclusion [29, 30, 37, 38, 41, 42]. In all of these studies, the diagnosis was determined using ADIS [21]. The rest of the studies relied on parents providing an accurate report of clinically significant anxiety symptoms or social difficulties. A summary of study characteristics can be found in Table 1.

**Table 1:** A summary of study characteristics

Source	Population	No. of participants	Age range^a^	Intervention	Delivery	Parental/caregiver involvement	Comparison	Outcome measures	SMD
Chalfant et al., 2007 [30]	Children with HFAD or Asperger disorder and a primary anxiety disorder.	47 (35 male, 12 female)	8-13 years (mean 10.8, SD 1.35)	CBT (n = 28)	Group sessions (6-8 children/group).	-	WL/TAU (n = 19)	SCAS-P	4.27
								SCAS-C	2.64
McConachie et al., 2013 [37]	Children with ASD and at least one anxiety disorder.	32 (28 male, 4 female)	9-13 years and 11 months	CBT (n = 17)	Group sessions (no./group not reported).	Separate parallel groups for parents.	WL/TAU (n = 15)	ADIS-C	0.43
								SCAS-P	0.20
								SCAS-C	0.04
McNally Keehn et al., 2013 [38]	Children with an ASD and at least one primary anxiety disorder of SAD, GAD or SoP.	22 (21 male, 1 female)	8-14 years (mean 11.26, SD 1.53)	CBT (n = 12)	Individual sessions.	-	WL/TAU (n = 10)	ADIS-P	1.35
								SCAS-P	0.91
								SCAS-C	0.47
Reaven et al., 2012 [39]	Children with ASD and clinically significant anxiety symptoms.	50 (48 male, 2 female)	7-14 years (mean 10.4 years, SD 1.7)	CBT (n = 24)	Children only group sessions (3-6 children/ group), parent and children sessions, parent-child dyad sessions.	Parent and children group sessions, parent only and child only group sessions, parent-child dyad sessions.	WL/TAU (n = 26)	ADIS-P	0.60
Schohl et al., 2013 [43]	Adolescents with ASD and social problems.	63 (47 male, 11 female, 5 not specified)	11-16 years (mean 13.65 years, SD 1.50)	Social skills intervention (n = 34)	Group sessions (≤10 adolescents per group).	Separate parallel groups for parents.	WL/TAU (n = 29)	SIAS	0.16
Sofronoff et al., 2005 [32]	Children with AS and anxiety symptoms.	71 (62 male, 9 female)	10-12 years	CBT Child only (n = 23). CBT Child + parent (n = 25)	Group sessions (3 children/ group, allocated by age and sex, with girls grouped together)	Separate parallel groups for parents (12-13 parents/group)	WL/TAU (n = 23)	SCAS-P	0.09
Storch et al., 2013 [29]	Children with ASD and a primary diagnosis of SAD, GAD or OCD.	45 (36 male, 9 female)	7-11 years	CBT (n = 24)	Individual sessions.	Separate parallel parent sessions +/- parental involvement in child-focused components.	WL/TAU (n = 21)	PARS	1.38
								MASC	0.48
								RCMAS	0.26
Sung et al., 2011 [40]	Children and adolescents with ASD and anxiety-related issues.	70 (66 male, 4 female)	9-16 years	CBT (n = 36)	Group sessions (3-4 participants/ group).	-	Social recreational group program (n = 34).	SCAS-C	0.07
White et al., 2013 [41]	Children with ASD and at least one of SoP, GAD, SP, or SAD.	30 (23 male, 7 female)	12-17 years (mean 14.58, SD 15 years)	CBT (n = 15)	Individual and group sessions (no./group not reported).	Parent education and coaching.	WL/TAU (n = 15)	PARS	0.32
								CASI Anx	0.37
Wood et al., 2009 [42]	Children with ASD and SAD, SoP or OCD.	40 (27 male, 13 female)	7–11 years, mean 9.20, SD 1.49	CBT (n =17)	Individual sessions.	60 min of each session spent with parents/family.	WL/TAU (n = 23)	ADIS-C/P	2.47
								MASC-P	1.21
								MASC-C	-0.03

*SMD* Standardised mean difference, *HFAD* High Functioning Autistic Disorder, *SAD* Separation anxiety disorder, *GAD* Generalised anxiety disorder, *SoP* Social phobia, *SP* Specific phobia, *OCD* Obsessive Compulsive Disorder, *CBT* Cognitive behavioural therapy, *WL/TAU* Wait list/Treatment as usual, *SCAS-C/P* The Spence Children’s Anxiety Scale – Child/Parent, *ADIS C/P* The anxiety Disorders Interview Schedule – Child/Parent, *SIAS* The social Interaction Anxiety Scale, *PARS* The Pediatric Anxiety Rating Scale, *MASC* The Multidimensional Anxiety Scale for Children, *RCMAS* The Revised Children's Manifest Anxiety Scale, *CASI*-*Anx* Child and Adolescent Symptom Inventory-4 *ASD* Anxiety Scale

^a^Mean and SD reported when data was available

### Risk of bias within studies

#### Selection bias – random sequence generation and allocation concealment

Six of the included studies performed adequate random sequence generation, either manually or generated by a computer [29, 37, 39–42]. Chalfant et al, Sofronoff et al, McNally Keehn et al, and Schohl et al. provided insufficient information about the randomisation process to determine the extent to which this may have affected the bias of these studies [30, 32, 38, 43]. McConachie et al. performed satisfactory and complete allocation concealment [37]. In contrast, Chalfant et al. and Sung et al. did not perform adequate allocation concealment [30, 40]. The remainder of the included studies indicated that allocation concealment was implemented, but did not provide sufficient information about the method of concealment [29, 32, 38, 39, 41–43].

#### Performance and detection bias – blinding of participants, personnel and outcome assessment

As previously stated, blinding of participants and personnel was not possible in any of the included studies. Furthermore, blinding of parent- and self-reported outcome measures was not feasible. In the studies conducted by Storch et al., McConachie et al., McNally Keehn et al., White et al., and Wood et al., clinicians rating the ADIS or PARS were blind to treatment allocation and these outcome measures were therefore considered to have a low risk of detection bias [29, 37, 38, 41, 42].

#### Attrition and reporting bias – incomplete outcome data and selective outcome reporting

Reaven et al. and Schohl et al. were thought to have a high risk of attrition bias since missing data was removed from the study analysis [39, 43]. The remainder of the included studies were deemed to have complete outcome data [29, 30, 32, 37, 38, 40–42]. There was no evidence of selective outcome reporting in any of the included studies [29, 30, 32, 37–43].

### Clinician reported outcome measures

**Fig. 2:**
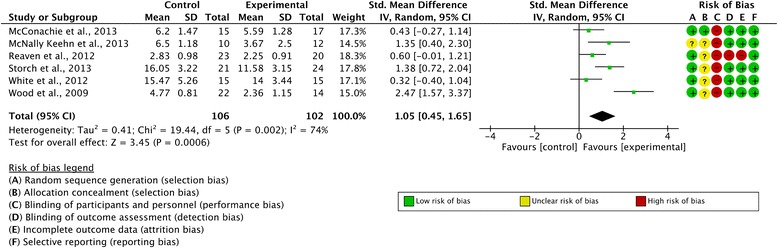
Forest plot of clinician-reported outcome measures with risk of bias summary

Six studies involving a total of 208 patients (102 in the treatment condition and 106 in the control condition) reported a clinician-reported outcome measure. All studies reported greater improvements post-treatment in the intervention condition compared to the control condition. The overall SMD was d = 1.05 (95 % CI 0.45, 1.65; z = 3.45, p = 0.0006) which can be considered a large effect. Based on these measures, the anxiety levels in the intervention groups were significantly lower than those seen in the WL/TAU groups at post-treatment. Considerable heterogeneity across the studies was detected (I^2^ = 74 %). A forest plot illustrating these results is included as Fig. 2.

### Parent reported outcome measures

**Fig. 3:**
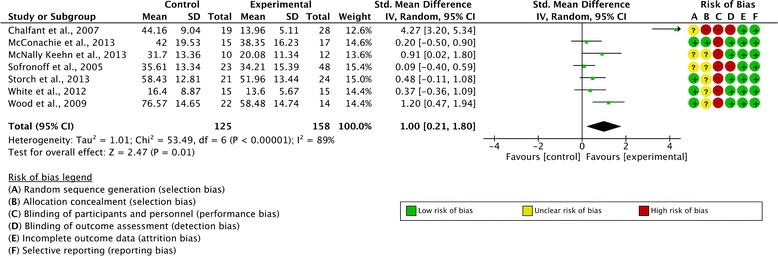
Forest plot of parent-reported outcome measures with risk of bias summary

Seven studies reported at least one of the selected parent-reported outcome measures. These studies involved 283 participants (158 in the experimental condition and 125 in the control condition). The overall SMD was d = 1.00 (95 % CI 0.21, 1.80; z = 2.47, p = 0.01) and the difference between the intervention and control conditions at post-treatment reached significance. There was significant heterogeneity across the included studies (I^2^ = 89 %). A forest plot illustrating these results is included as Fig. 3. The SMD reported by Chalfant et al. [30] was considered an outlier since it was substantially higher than the ones reported in the other studies. Once this outlier was removed, the overall SMD decreased to 0.48 (95 % CI 0.14, 0.82; z = 2.80, p = 0.005). Although the summary estimate decreased following removal of the outlying study, the treatment effect remained significant.

### Self-reported outcome measures

**Fig. 4:**
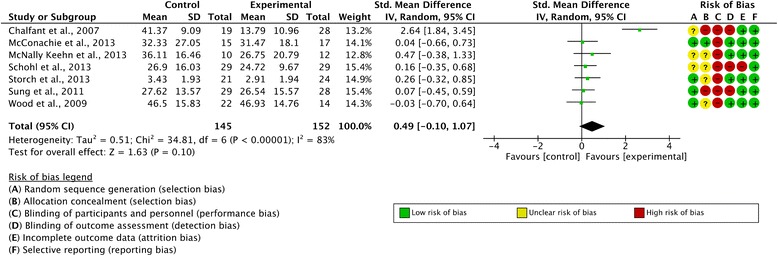
Forest plot of self-reported outcome measures with risk of bias summary

Self-reported outcome data from 297 participants (152 in the intervention condition and 145 in the control condition) across seven studies was reported. The overall SMD was d = 0.649 (95 % CI -0.10, 1.07; z = 1.63, p = 0.10) with no significant difference between the experimental and control conditions at post-treatment. There were high levels of heterogeneity across the studies (I^2^ = 83 %). A forest plot illustrating these results is included as Fig. 4. Once more, the SMD reported by Chalfant et al. [30] was an outlier and removal of this study reduced the overall SMD to 0.14 (95%CI -0.11, 0.39; z = 2.80, p = 0.005). A sensitivity analysis was carried out by removing Schohl et al. [43], the only study that did not use a CBT intervention. This changed the overall SMD to 0.55 (95 % CI -0.16, 1.27; z = 1.51, p = 0.13) and the difference between intervention and control conditions at post-treatment remained insignificant.

### Group versus individual intervention

A subgroup analysis comparing individual +/- group therapy versus group therapy only was conducted for clinician- and parent-reported outcome measures. For clinician-reported outcome measures, the summary estimate for studies that used individual +/- group therapy was 1.70 (95 % CI 1.01, 2.40; z = 3.37, p = 0.0007). This can be compared to the summary estimate for studies that used group therapy only which was 0.47 (95 % CI 0.08, 0.86; z = 2.36, p = 0.02). Although the difference between experimental and control conditions at post-treatment were significant for both individual +/- group therapy and group therapy only, the confidence intervals for the two subgroups did not overlap. This indicates that, according to clinician-reported outcome measures, individual therapy as part of the intervention improved its effectiveness.

For parent-reported outcome measures, the summary estimate was 0.81 (95 % CI 0.36, 1.26; z = 3.56, p = 0.0004) for studies that used individual +/- group therapy and 1.17 (95 % CI -0.28, 2.62; z = 1.58, p = 0.11) for those that used group therapy only. The confidence intervals clearly overlapped and it can therefore be concluded that, for parent-reported outcome measures, individual therapy components did not significantly increase the effectiveness of the interventions. However, it should be noted that the summary estimate for the studies that used individual +/- group therapy reached significance and for those that used group therapy only failed to do so. This implies that individual therapy modules may be necessary to make therapy beneficial.

### Follow-up data

McConachie et al. [37], McNally Keehn et al. [38], Sofronoff et al. [32], Storch et al. [29], and Sung et al. [40] reported follow-up data. The follow-up results were obtained between 6 weeks and 6 months after the end of the intervention. In three studies, there were only follow-up data available for the intervention condition since it was considered unethical to withhold treatment from the WL/TAU group. Therefore, only follow-up data from the intervention groups were examined in this review. The results in all five studies showed no statistically significant (p < 0.05) difference between the post-treatment and follow-up scores for any of the outcome measures. Although the sample of studies that reported follow-up data was small, the results indicate that the positive effects of the intervention were maintained for some months after the final session.

## Discussion

### Summary of evidence

This meta-analysis aimed to investigate the effectiveness of psychosocial interventions for reducing anxiety in children and adolescents with ASD. Clinician- and parent-reported outcome measures showed that psychosocial interventions were superior to WL/TAU control conditions at post-treatment. In the parent-reported outcome measures category, the SMD from Chalfant et al. [30] was identified as an outlier. Although the summary estimate for parent-reported outcome measures decreased following removal of this study, the post-treatment difference between the experimental and control groups remained significant. It should also be noted that Chalfant et al. [30] was rated as having a high risk of bias in multiple domains of the risk of bias assessment due to inadequate random sequence generation, allocation concealment, and blinding of participants, personnel and outcome assessment. For self-reported outcome measures, the difference between experimental and WL/TAU groups at post-treatment failed to reach significance. The subgroup analysis used to compare individual and group delivery of treatment showed that individual +/- group therapy was more effective than group treatment alone, particularly for clinician-reported outcome measures. The results from this meta-analysis indicate that the evidence for using psychosocial interventions to manage anxiety in ASD youth is fairly robust, but that the degree of effect differs across anxiety informants.

### Comparison of results to current literature

A meta-analysis of RCTs investigating the effectiveness of CBT as a treatment for anxiety disorders in children and adolescents with ASD was published by Sukhodolsky et al. shortly after the study design for the present review had been established [45]. Sukhodolsky et al. reported a treatment effect of 1.21 (95 % CI 0.50, 1.97) for clinician-reported outcome measures, 1.19 (95 % CI 0.23, 2.14) for parent-reported outcome measures and 0.68 (95 % CI -0.17, 1.54) for self-reported outcome measures. In addition to the studies included in the meta-analysis conducted by Sukhodolsky et al., the present study analysed two supplementary studies – one that was published in late 2013 [37] and one that used a social skills intervention rather than CBT [43]. The addition of these studies consistently reduced the overall SMD and narrowed the 95 % CI, which may indicate that the treatment effect reported by Sukhodolsky et al. may have been overestimated.

### Treatment modality

CBT modified for children and adolescents with ASD was the primary intervention modality studied in this meta-analysis. However, the search was extended to other types of psychosocial interventions since some of these have been shown to be as effective as CBT in ASD youth [14, 40]. The only other type of psychosocial intervention that was identified in the search was a social skills intervention. The association between social disability and anxiety in ASD youth seems to be bidirectional. A higher IQ and greater social impairment has been shown to be associated with more severe anxiety symptoms, potentially due to the fact that individuals with higher IQ are more aware of their social deficits [23]. On the other hand, anxiety disorders can increase social isolation and disability [15]. Components of social skills interventions are essential as part of any psychosocial intervention aimed at ASD youth and a social skills approach to managing anxiety in ASD youth was therefore considered valid for inclusion in this meta-analysis.

Although psychosocial interventions seem to be effective in reducing anxiety compared to WL/TAU control conditions, the literature involving an active control is limited. Sung et al. [40] compared the effectiveness of CBT and a social recreational group and found no significant difference between the interventions and active control conditions at post-treatment and at 3- and 6-month follow-up. Russell et al. [14] also failed to detect a significant difference between the effectiveness of CBT and an anxiety management intervention on OCD symptoms in adults with ASD. Neither of these studies included a WL/TAU condition. Although these studies indicate that a variety of psychosocial interventions may be equally effective in reducing anxiety symptoms in the paediatric ASD population, further research with more homogenous samples and multiple treatment arms will be necessary to fully establish the most effective treatment modalities.

### Individual versus group intervention

Psychosocial interventions for ASD youth can be delivered in a variety of formats. Studies have been conducted using interventions in a group or individual format (or a combination of the two), but no study has yet compared these delivery methods against one another [6]. Individual therapy may be more flexible and allow treatment to be designed according to the individual’s needs. Group therapy, on the other hand, may be beneficial in terms of peer support and sharing of experiences. The results from the subgroup analysis performed in this meta-analysis indicate that individual +/- group therapy is more beneficial than group therapy alone. For clinician-reported outcome measures, there was a significant difference between intervention and control conditions at post-treatment for both individual and group interventions. However, the summary estimate was significantly higher for the studies that used individual therapy only. For parent-reported outcome measures, the difference between intervention and control conditions was significant in those studies that used individual +/- group therapy. In contrast, in the studies that used group therapy only, the difference between intervention and control conditions failed to reach significance.

### Modifications

Adapting psychosocial intervention programs to the needs of children and adolescents with ASD may improve the delivery of and response to treatment [46]. These modifications aim to minimise potential barriers that may limit the efficacy of treatment, including reduced ToM abilities, cognitive inflexibility, executive function deficits and concrete thinking [47]. The modification trends for CBT programs include the use of visual aids, incorporation of child-specific interests into the intervention, using highly structured sessions, and having a flexible number and length of sessions [46, 47].

Parental involvement in psychosocial interventions aimed at reducing anxiety in the paediatric population has been shown to positively affect treatment outcome in both typically developing children [48] and children with Asperger syndrome [41, 49]. Further studies have suggested that parenting and family factors, such as family accommodation, are associated with treatment outcomes [14, 30]. It is important to consider the potential benefits of parental engagement in therapy targeted at the ASD population. Firstly, if parents are taught to become co-therapists, there may be reinforcement and generalisation of the taught skills in the home environment [32]. Secondly, involvement of parents may reduce parental anxiety and provide the necessary communication and coping skills to reduce the child’s anxiety [40].

### Self-reported outcome measures

Self-reported outcome measures are used as they are thought to provide reliable information about an individual’s health and illness [50]. The use of self-reported measures in the paediatric population has become increasingly common. However, research of its efficacy is lacking [51]. In this review, parent- and clinician-reported outcome measures showed a greater treatment effect than self-reported measures which failed to reach significance. This finding mirrors results from recent studies of multiple informant agreement on anxiety measures in typically developing children [16] and children with ASD [52].

The reporting trends of different anxiety informants may reflect an actual difference in the perceived effects of the treatment between clinicians, parents and children. However, this inconsistency may be caused by other factors, which makes the use of self-reported outcome assessment questionable in paediatric populations. Children may lack the general cognitive abilities, self-awareness and understanding of health-related concepts to accurately report these types of outcome measures [53]. Furthermore, there is a general lack of understanding of how heath and illness perceptions are expressed by children at different stages of development [54].

In the paediatric ASD population, the use of self-reported measures can be especially challenging [11]. A proportion of the ASD population do not have the necessary communication and cognitive skills to conceptualise their thoughts, emotions and behaviours, as well as identify internal states such as worry and fear [11, 49]. Furthermore, individuals with ASD may not be able to distinguish between their feelings of anxiety from their experience of core ASD symptoms [7]. The anxiety symptoms may therefore be reported inaccurately. Measures of anxiety reported by primary caregivers are often considered more reliable in ASD youth [49]. However, if the child lacks expressive language skills, even caregivers may struggle to separate anxiety and ASD symptomology [11].

### Choice of outcome measures

The presentation of anxiety in the paediatric ASD population is fundamentally different from the anxiety seen in typically developing children and there is a general lack of means to separate anxiety from core ASD symptomology [10]. Therefore, outcome measure tools developed to measure anxiety in typically developing children may not be adequate at capturing the unique nature of anxiety in an ASD population. Furthermore, the definition of a successful outcome in the anxious ASD population may include components that are not relevant for typically developing children, such as social and communication skills, and level of participation [11].

All the outcome measures selected for this review were developed to measure anxiety in typically developing children. There is some evidence that SCAS-C/P, SCARED-C/P, CASI, MASC-P, PARS and ADIS-C/P can be used to accurately measure anxiety in ASD. There is, however, some disagreement between studies regarding which instruments have the most robust measurement properties in the paediatric ASD population [10, 11]. Validated and sensitive instruments modified for use in children and adolescents with ASD will be necessary to improve comparability between study results as well as progress in treatment development [11]. However, until further psychometric research is carried out, the measure of anxiety in children and adolescents with ASD will rely on instruments developed for typically developing children.

### Future research

Future research with larger sample groups and extended follow-up will be necessary to determine the precise effectiveness of psychosocial interventions for reducing anxiety in the ASD youth. Additional qualitative studies as well as the use of active control groups will be necessary to understand the specific components of psychosocial interventions that are most effective. More experience is required to establish empirically validated treatment manuals for use specifically in children and adolescents with ASD and find ways in which these manuals can be modified for different developmental stages. Future research will also have to ascertain how psychosocial interventions can be modified for lower functioning children and adolescents with ASD. Finally, further development of outcome measures will be essential to produce more reliable results.

### Limitations

The results of this review may not be generalisable to the paediatric ASD population due to small samples, exclusion of the lower functioning subsets of the spectrum as well as limited representation of females and ethnic minorities. Although blinding of participants, personnel and most outcome assessments was not feasible, this lack of blinding may bias the results (perhaps due to wishful thinking and social pressure). It should also be noted that the participants in these studies were self-selected and therefore could have been drawn from an unusually motivated population.

The main limitation of this review was the inclusion of a relatively small number of studies due to the limited research in this area. This made it impossible to statistically analyse certain aspects of the data, including publication bias. Further limitations included clinical and methodological variability between studies. This variability made it impossible to conduct meaningful subgroup analyses to assess the potential effects of age, anxiety diagnoses, parental involvement and other factors on the treatment effect. In addition, there was a lack of studies in languages other than English. Finally, although data extraction and study analysis was conducted by two independent reviewers using a well validated and structured manual, there was still a certain level of subjectivity to the review process.

## Conclusion

This meta-analysis aimed to systematically evaluate and summarise the evidence for using psychosocial interventions to manage anxiety in children and adolescents with ASD. Clinician- and parent-reported outcome measures showed that psychosocial interventions were superior to WL/TAU control conditions at post-treatment and individual therapy seemed to be more effective than group treatment. For self-reported outcome measures, the difference between experimental and WL/TAU groups at post-treatment failed to reach significance. However, more methodologically rigorous research will be necessary to ascertain the precise potential of psychosocial interventions to reduce anxiety in the paediatric ASD population.

## Abbreviations

ADIS-C/P: Anxiety Disorders Interview Schedule – Child/Parent version
ADOS: Autism Diagnostic Observation Schedule
AS: Asperger syndrome
ASD: Autism spectrum disorder
CASI-Anx: Childhood Anxiety Sensitivity Index - Anxiety
CBT: Cognitive behavioural therapy (CBT)
CCDEF: Cochrane Collaboration Data Extraction Form
HFAD: High-Functioning Autistic Disorder
MASC-C/P: Multidimensional Anxiety Scale for Children – Child/Parent version
OCD: Obsessive compulsive disorder
PARS: Pediatric Anxiety Rating Scale
RCMAS: Revised Children's Manifest Anxiety Scale
RCT: Randomised Control Trial
SCAS-C/P: Spence Children’s Anxiety Scale – Child/Parent version
SIAS: Social Interaction Anxiety Scale
SMD: Standardised mean difference
TAU: Treatment-as-usual
ToM: Theory of Mind
WL: Waitlist
